# Differences in microbial composition and salt tolerance between rhizosphere and bulk soil of *Kalidium foliatum*

**DOI:** 10.3389/fpls.2026.1920612

**Published:** 2026-10-07

**Authors:** Lamei Jiang, Deyan Wu, Eryang Li, Wenjing Li, Yuehan Liu, Xiaotong Li, Guang Yang, Guanghui Lv

**Affiliations:** 1 College of Life Sciences, Xinjiang Agriculture University, Urumqi, China; 2 College of Ecology and Environment, Xinjiang University, Urumqi, China; 3 Farmland Irrigation Research Institute, Chinese Academy of Agricultural Sciences, Xinxiang, China

**Keywords:** functional genes, ion transport, permeation regulation, rhizosphere microorganisms, salt stress

## Abstract

Salt is an important abiotic stress that affects plant growth. Research has shown that plants can recruit beneficial microorganisms from the surrounding soil. However, the ecological functions of the rhizosphere microbiome in salt tolerant plants have not been explored to a large extent. Based on this, this study took the halophyte *K. foliatum* as the research object, and used metagenomics technology to analyze the differences in microbial composition and salt tolerance functional genes between the rhizosphere and bulk soil of *K. foliatum* in different salt habitats, providing new theoretical basis for salt alkali management. The results showed that the dominant groups in the rhizosphere soil microbial community of *K. foliatum* under two salt habitats were Actinobacteria, Proteobacteria, Bacteroidetes, and Chloroflexi. The abundance of 49 genera in Actinobacteria in high salt rhizosphere were significantly higher than that in low salt rhizosphere. These microorganisms belong to soil salt tolerant and halophilic microorganisms, and have the ability to improve plant salt tolerance. In high salt habitat, the expression of genes related to ion transport (*nqrA*, *nhaC*, *rnfC*, *chaA*, and *yrbG*) and the synthesis of osmoregulatory substances (*proP*, *ectA*) was significantly increased. These genes are associated with Na^+^, K^+^, Mg^2+^, proline and ectoine involved in transport or synthesis. The correlation indicates that halophilic archaea have a greater impact on functional genes than other microorganisms, suggesting that halophilic bacteria have a more prominent ability to regulate salt tolerance of *K. foliatum*.

## Introduction

Soil microorganisms play an important role in plant resistance to stress, such as improving plant salt tolerance by regulating the ion homeostasis of plant roots, producing plant hormones, promoting the accumulation of root osmotic substances, enhancing plant antioxidant activity, and increasing nutrient absorption. As a key driving force of plant productivity, the important role of soil microorganisms in saline soil needs to be fully understood and revealed, especially in low productivity desert ecosystems. How soil microorganisms respond and adapt to changing soil salinization conditions has also become a hot topic in the field of environmental microbiology in recent years (Lanza et al., 2019; Rath et al., 2019; Sun et al., 2022). Soil microorganisms also play an important role in maintaining ecosystem stability, reflecting the evolution of soil quality and serving as one of the indicators of ecosystem health (Diacono and Montemurro, 2010). The latest advances in the field of molecular ecology, especially the development of metagenomics technology, can provide new insights into the changes in microbial taxa and functions under salt stress.

The response mode of soil microbial diversity to salt changes is related to the environment in which it is located. Many researchers have explored the impact of salt on soil microbial community diversity and structure. Li et al. (2021) result showed that as salt stress increased, the diversity of rhizosphere bacteria in salt sensitive plants decreased, while the diversity of salt tolerant plants increased. Maintaining a diverse microbial community in salt tolerant plants may play an important role in plant salt tolerance. As the salt content increases, high salt content increased the relative abundance of Bacteroidetes and Firmicutes, while reducing the relative abundance of Acidobacterium, Chloroflexi, and Cyanobacteria (Zhang et al., 2020). Rath et al. (2019) found that the number of OTUs in Gamma Proteobacteria and Bacteroidetes was positively correlated with high salt tolerance. In *Medicago truncatula*, salt stress leads to changes in bacterial populations, particularly an increase in the abundance of *Enterobacter* (Yaish et al., 2016). Many previous studies have shown that a large number of microorganisms related to salt stress adaptation are enriched in the rhizosphere of salt tolerant plants. For example, Qin et al. (2016) found that the core rhizosphere bacterial pseudomonas species of halophilic plants can improve salt tolerance in non-halophilic plants. Xiong et al. (2020) revealed that *Bacillus flexis* KLBMP 4941 recruited from coastal halophytes can promote the growth of host plants under salt stress.

In recent years, research on the rhizosphere microbiome and metagenomics of halophilic plants has significantly advanced our understanding of the response of microbial communities to salinity, but the research content still needs to be further explored. Although Tang et al. (2023) used shotgun metagenomics to study *Suaeda glauca* and *S. salsa* along the soil root continuum, they did not specifically investigate genes related to ion transport and osmotic regulation in salt tolerant plants. Its functional analysis focuses on a wider range of metabolic categories rather than salt tolerance specific pathways. Tugbaeva et al. (2025) used 16S rRNA metagenomic sequencing to study the bacterial community of the halophyte *Spergularia Marina* in low and moderate sulfate chloride salinity habitats, identifying 55 phyla and 309 genera, with Proteobacteria and Bacteroidetes being dominant groups. They found that the alpha diversity of bacteria in bulk rhizosphere soil decreased with increasing salinity, while in rhizosphere soil it increased. The proportion of salt tolerant genera such as *Flavobacterium*, *Alteromonas*, *Bacillus*, and *Salinmicrobium* increased with increasing salinity. However, this study relies on 16S rRNA amplicon sequencing and cannot reveal the functional gene profile or specific molecular mechanisms of salt tolerance.

There are abundant salt tolerant microorganisms in the rhizosphere of *K. foliatum*, which can improve plant growth conditions and enhance plant tolerance to environmental stresses such as salt alkalinity and drought (Wang et al., 2023). Research has found that there are significant differences in the metabolic activity and microbial community composition of rhizosphere microorganisms in *K. foliatum* under different NaCl concentration cultivation conditions, with the highest metabolic activity observed at 5% NaCl (Chen et al., 2019). Zhang et al. (2021) isolated 21 species belonging to 12 genera and 4 phyla from the rhizosphere microorganisms of *K. foliatum*. Most of the strains have rhizosphere growth promoting effects, among which genera such as *Isoptericola* and *Sinomicrobium* have rarely been reported before, suggesting that salt plants contain abundant undeveloped rhizosphere growth promoting microorganisms, which needs further exploration. While these studies have established that *K. foliatum* harbors abundant salt-tolerant and plant growth-promoting microorganisms, they share a critical limitation: all are based on either cultivation-dependent isolation or amplicon-based taxonomic profiling, which cannot reveal the functional gene repertoire: the actual metabolic pathways and molecular mechanisms, through which these microorganisms contribute to host salt tolerance. Moreover, previous studies have typically examined a single salinity level or used artificial salt treatments in laboratory settings, leaving the functional gene-level responses of the rhizosphere microbiome to naturally occurring salinity gradients largely unexplored. Based on the above, this study aims to analyze: How do microbial community composition and salt tolerance genes respond to changes in salinity gradients in the rhizosphere soil of the halophyte *K. foliatum*, and through which ecological and metabolic mechanisms do they enhance the host plant’s salt tolerance. And through this research, explore salt tolerant microorganisms and provide theoretical basis for the management and development of saline soil in Xinjiang.

## Research methods

### Study site description, sample plot layout and collection

The Ebinur Lake Wetland National Nature Reserve(ELWNNR) in Xinjiang (44°43′N-45°12′N, 82°35′E-83°40′E) is the lowest depression and water salt concentration center in the southwest margin of the Junggar Basin (Yang et al., 2022). The climate in this area belongs to the temperate continental arid climate. According to the previous sampling method, high salt and low salt habitat were selected as study sites near the Aqikesu River in ELWNNR (Jiang et al., 2024). The soil salt concentrations were as follows: 10.15 ± 0.07 g/kg(A) and 3.62 ± 0.08 g/kg(B)(**Figure 1**).

**Figure 1:**
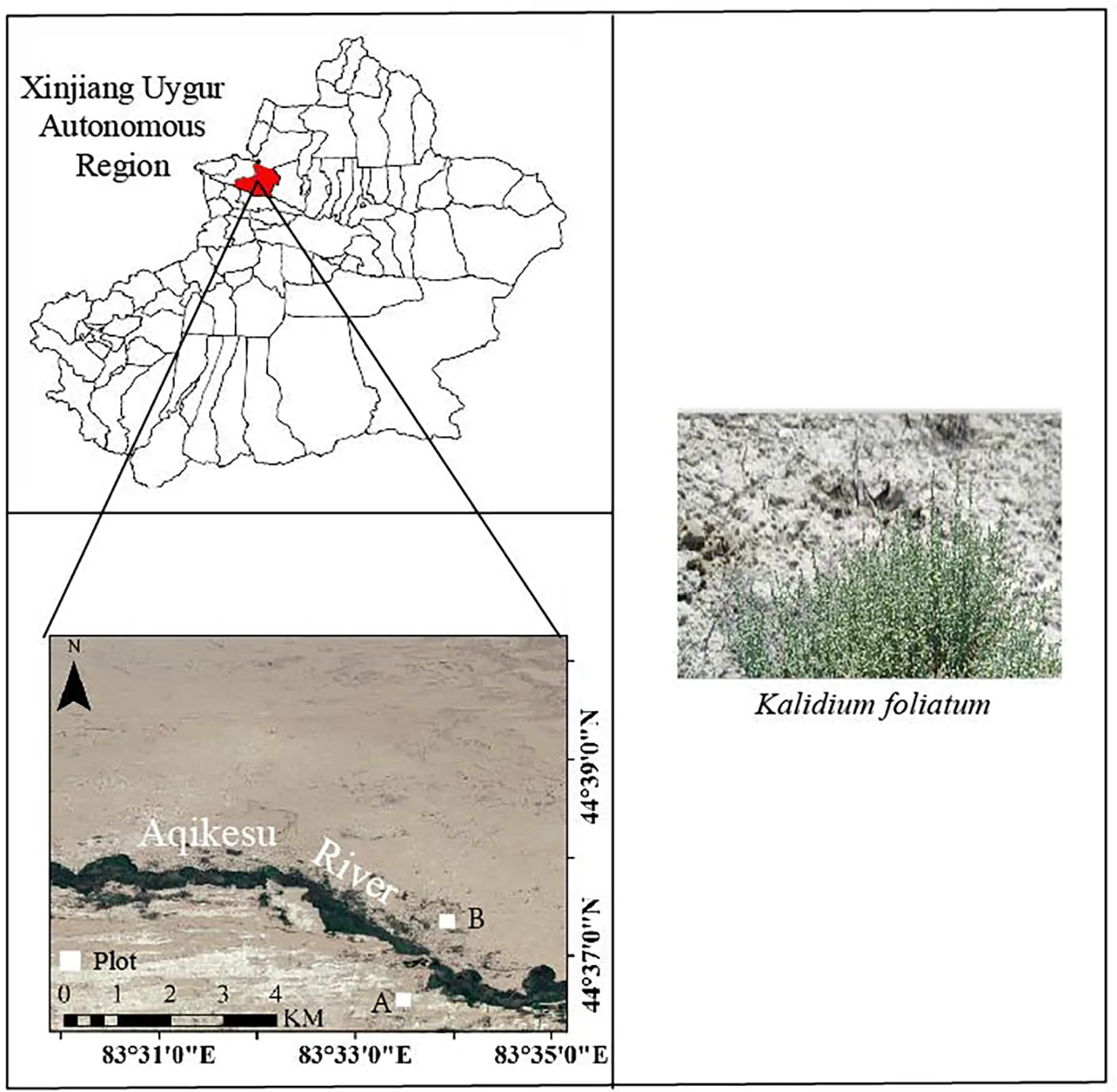
Schematic diagram of plot layout and plant morphology. Soil sampling: Rhizosphere soil sampling: In order to make the collected samples more representative, 18 plants were collected from each plot, and the soil of three plants was combined into one soil sample. Six rhizosphere soil samples were taken from each salt habitat. The roots of the plants were dug out, the soil attached to the roots was shaken off, and the roots were stored in sterile bags at low temperatures. They were immediately taken back to the laboratory and washed with PBS solution to remove the soil from the roots as rhizosphere soil. Bulk soil that was originally far away from the plants, a sterile shovel was used to dig a pit of 0–40 cm, and samples were taken at the profile. The soil samples were loaded into a 50 mL sterilized centrifuge tube as bulk soil. Six bulk soil samples were collected from each salt habitat, for a total of 24 mixed soil samples. Physical and chemical properties of rhizosphere soil: The remaining part of the collected rhizosphere soil was filtered with sterile high-purity water and dried in an aluminum box for later use. Treatment of rhizosphere soil microbial assay: Conducted in a laboratory sterile operating table, each root sample is immediately placed into a sterile bottle and the root surface is cleaned with PBS buffer. Filter with sterile gauze into a 50 ml sterile centrifuge tube, centrifuge at 10000 g for 1 minute using a low-temperature centrifuge, and concentrate the rhizosphere sample. Store the concentrated sample in an ultra-low temperature freezer at -80 °C for metagenomic sequencing.

### Soil chemical and physical properties

soil salinity content (SA) and using the weighing method The soil organic carbon (SOC), total nitrogen (TN), were determined using the potassium dichromate dilution heat method and Kjeldahl digestion method We determined the above physicochemical properties using soil agrochemical analysis (Bao, 2000).

### Soil microbial determination

#### DNA extraction, library construction, and metagenomic sequencing

The soil DNA of the halophytic shrubs was extracted from 0.2 g soil using the OMEGA Soil DNA Kit (Omega, Bio-Tek, Norcross, GA, USA). Qubit 2.0 was used to quantitatively detect DNA sample concentration, and then using bridge PCR, construct PE library. Then perform 12 cycles of bridge amplification. Obtain DNA clusters and load them into Illumina cBot. After quantitative and library detection using Thermo Qubit 4.0, the constructed library was sequenced and analyzed. Paired end sequencing was performed using Illumina Genome Analyzer (HiSeq PE150, Illumina Inc., San Diego, CA, USA), Sangon Co., Ltd. (Shanghai, China).

#### Sequence assembly and gene prediction

The study used Fastp software (https://github.com/OpenGene/fastp). Evaluate the quality of raw sequencing data. Remove low-quality base percentages (Q value<20), linker sequences with lengths less than 50 nucleotides, and their paired end reads to obtain relatively accurate data. Using assembly software IDBA-UD to assemble high-quality reads (Peng et al., 2012). Obtain contigs based on the overlapping relationship between reads. Next, conduct a comprehensive evaluation of the relevant contigs. Use Prodigal to predict the ORFs of contigs in the concatenated results. Select genes with nucleic acid lengths greater than or equal to 100bp and translate them into amino acid sequences. Due to the low microbial content in bulk soil caused by desert and salt factors, the four samples cannot meet the DNA quality requirements for sequencing. When analyzing metagenomic data, 20 soil samples were analyzed. In order to perform gene prediction for each sample, CD-HIT4.7 software was used to remove redundancy. Align the clean reads of each sample with non-redundant gene set sequences using Bowtie 2. Subsequently, the abundance of genes in each sample is determined based on the calculation of gene length.

#### Annotations on species and functions

Use BLAST (BLAST Version 2.2.28+) to compare the sequences in the non-redundant gene set with the NR database (BLAST comparison sets the expected value [E-value] to 1e-5) (Altschul et al., 1997). Using a taxonomic database paired with NR libraries, species are labeled for taxonomy, and then the relative species abundance is calculated based on the sum of gene abundance associated with each species. BLASTP uses the Kyoto Encyclopedia of Genes and Genomes (KEGG) database to compare sequences in non-redundant gene sets. Based on the comparison results, KOBAS 2.0 (KEGG Orthology Based Annotation System) was used for functional annotation (Xie et al., 2011).

### Data analysis

The formula for calculating microbial diversity is as follows:

Selecting Chao, Simpson, and Shannon indices to characterize microbial species diversity, the calculation formula is as follows:


$$\text{Chao}={\text{S}}_{\text{obs}}+\frac{{\text{n}}_{1}\left({\text{n}}_{1}-1\right)}{2\left({\text{n}}_{2}-1\right)}$$

Chao represents the estimated number of OTUs, while *S_obs_* represents the actual observed number of OTUs; n1 represents the number of OTUs containing only one sequence, and n2 represents the number of OTUs containing only two sequences.


$$\text{Simpson}=\frac{\sum_{\text{i}=1}^{{\text{S}}_{\text{obs}}}{\text{n}}_{\text{i}}\left({\text{n}}_{\text{i}}-1\right)}{\text{N}\left(\text{N}-1\right)}$$

$$\text{Shannon}=-\sum_{\text{i}=1}^{{\text{S}}_{\text{obs}}}\frac{{\text{n}}_{\text{i}}}{\text{N}}\ln\frac{{\text{n}}_{\text{i}}}{\text{N}}$$

Among them, *S_obs_* represents the actual number of observed OTUs; Ni is the number of sequences contained in the i-th OTU; N is the number of all sequences.

Analysis of differences in diversity index, functional gene abundance, and microbial abundance among different habitats.

Based on the *multicomp* package in R software, Shapiro Wilk and Levene’s methods were used to test the normality and homogeneity of variance of the data. Independent sample t-test (*P<* 0.05) was used to evaluate the significant differences between rhizosphere soil and bulk soil in the same location. The same independent sample t-test (*P<* 0.05) was used to analyze the differences in functional gene abundance and microbial abundance, applied multiple comparison corrections conducted FDR testing, to limit the proportion of errors that occur.

Analysis of the correlation between functional gene abundance and microbial abundance.

Based on correlation analysis, this study explores the correlation between the abundance of dominant phyla and salt tolerant functional genes in the rhizosphere and bulk soil of *K. foliatum*. Node and edge data were obtained using the R software “*igraph*” package, with Spearman’s correlation coefficient *r* > 0.70 and *P* < 0.01. The network was visualized using Cytoscape and Gephi software. Using the same method as above, analyze the correlation between the abundance of halophilic and salt tolerant microorganisms and salt tolerant functional genes.

## Results and analysis

### Microbial diversity and composition of *K. foliatum* soil in two salt habitats

The Chao index of soil microorganisms in the rhizosphere of *K. foliatum* showed that high salt habitat was greater than low salt habitat, while the Shannon index showed that low salt rhizosphere soil was significantly greater than low salt bulk soil. The Simpson index was slightly higher in low salt habitat than in high salt habitat (**Figure 2**).

**Figure 2:**
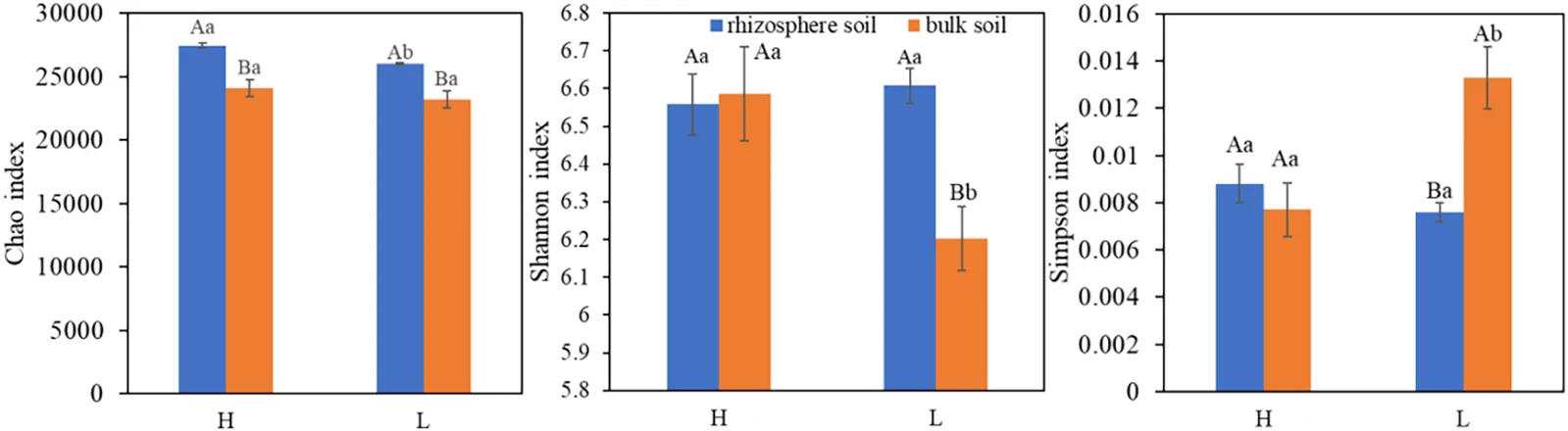
Differences in microbial diversity of *K. foliatum* rhizosphere and bulk soil under two saline habitats. The uppercase letters in the figure represent the differences in soil properties between rhizosphere and bulk soil in the same saline soil environment, while the lowercase letters represent the differences between high saline and low saline soil in the same type of soil (rhizosphere or bulk soil).

Under two salt habitats, the rhizosphere and bulk soil microorganisms of *K. foliatum* were mainly Actinobacteria and Proteobacteria, and the content of Proteobacteria in rhizosphere soil is higher than that in bulk soil, while Actinobacteria is more abundant in bulk soil (**Figure 3**).

**Figure 3:**
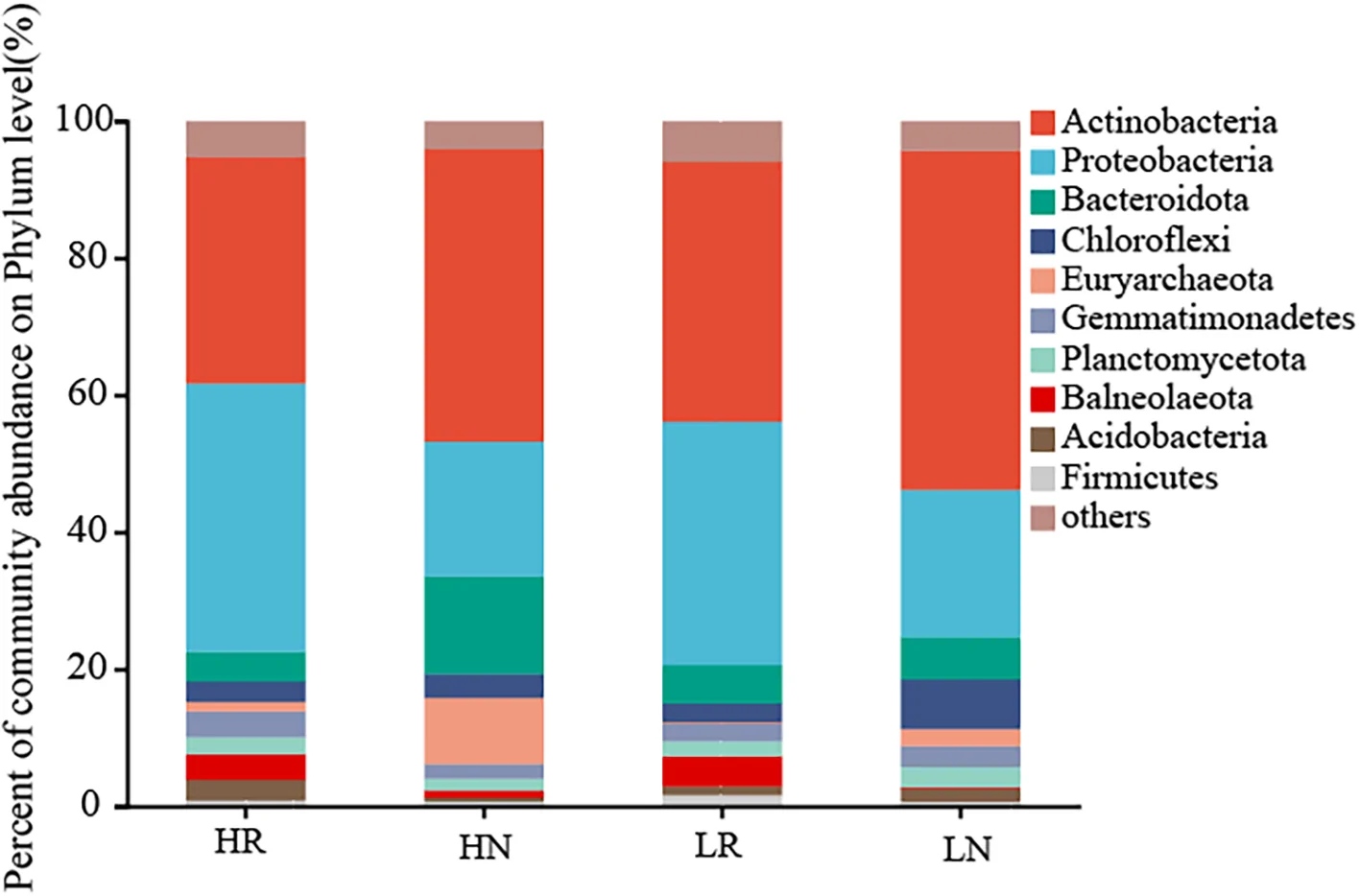
Microbial phylum composition of *K. foliatum* rhizosphere and bulk soil under two saline habitats. HR represents high salt rhizosphere soil, LR represents low salt rhizosphere soil, HN represents high salt bulk soil, and LN represents low salt bulk soil, the same as below.

### Differences in salt tolerance and halophilic microorganisms in *K. foliatum* soil under two salt habitats

The microbial abundance of 49 genera in the Euryarchaeota were significantly higher than that in the low salt rhizosphere soil, for example *Halomicroarcula, Natrialba, Halopiger* (**Figure 4**, **Supplementary Figures S1**, **S2**).

**Figure 4:**
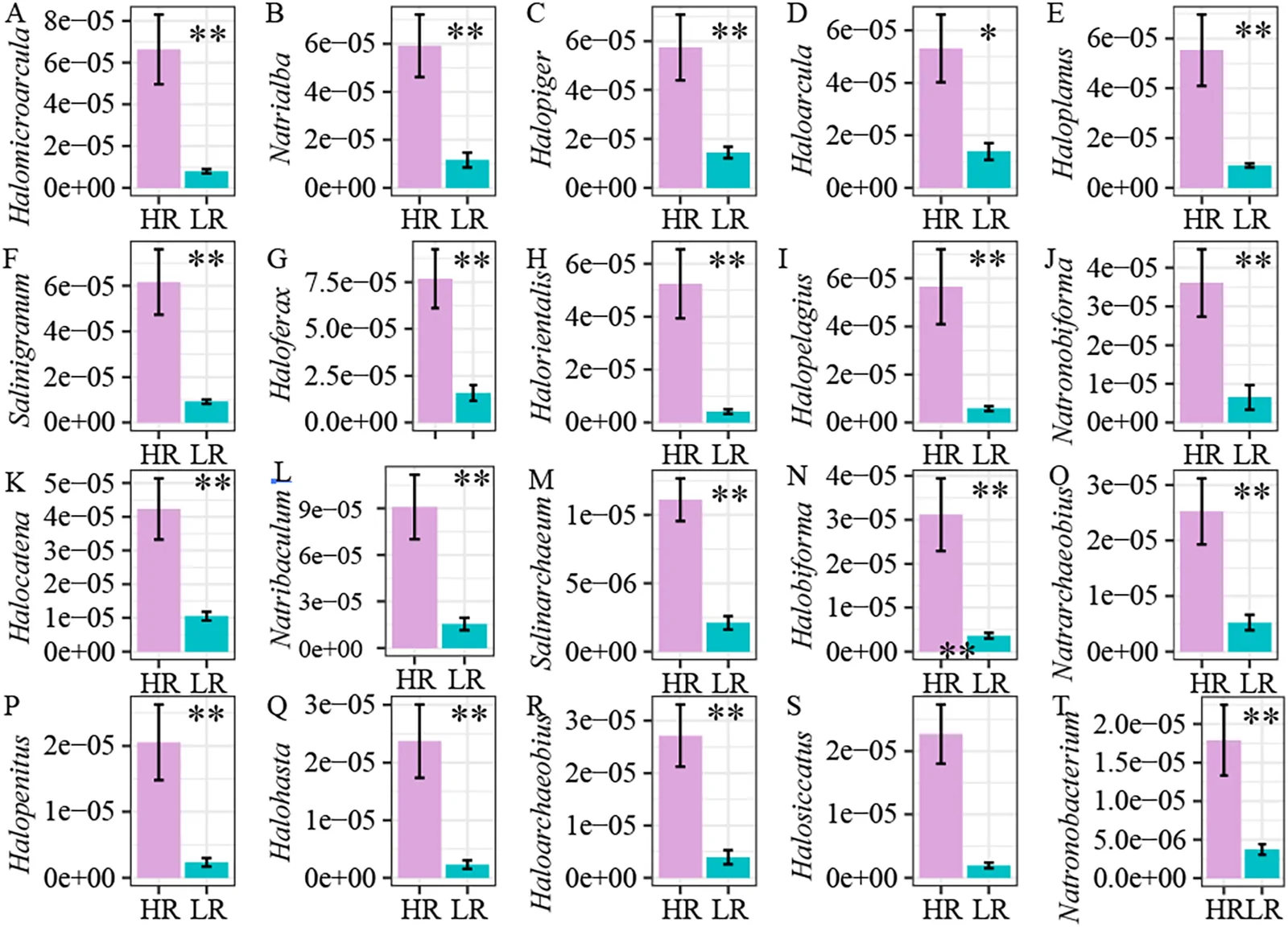
Differences in the abundance of halophilic archaea in the rhizosphere soil of *K. foliatum* under different habitats. * *P* < 0.05, ** *P* < 0.01.

### Differences in salt tolerant cycle functional genes involved by microorganisms

Microorganisms involved in H^+^/Na^+^transport (*ATPVA*, *ATPVD*, *ATPVI*, *rnfB*, *rnfC*), K^+^ absorption and transport (*kdpD*, *trkA*, *trkH*, and *K07222*), Ca^2+^/H^+^ reverse transport (*chaA)*, Mg^2+^ transport (*mgtE*), Na^+^/H^+^reverse transport (mnhB, mnhD), Na+transport (*nqrA*), sodium/proline co transporter (*putP*), and cation/H^+^ reverse transport (*yrbG*) were relatively abundant in high salt rhizosphere soil compared to low salt rhizosphere soil. In high salt bulk soil, the relative abundance of microbial ion transport gene *mnhF* was higher. *MnhG*, *nhaC*, *nqRA*, *nqRB*, *nqRC*, *nqRD*, and *yrbG* were all higher than those in the low salt bulk soil (**Figure 5**).

**Figure 5:**
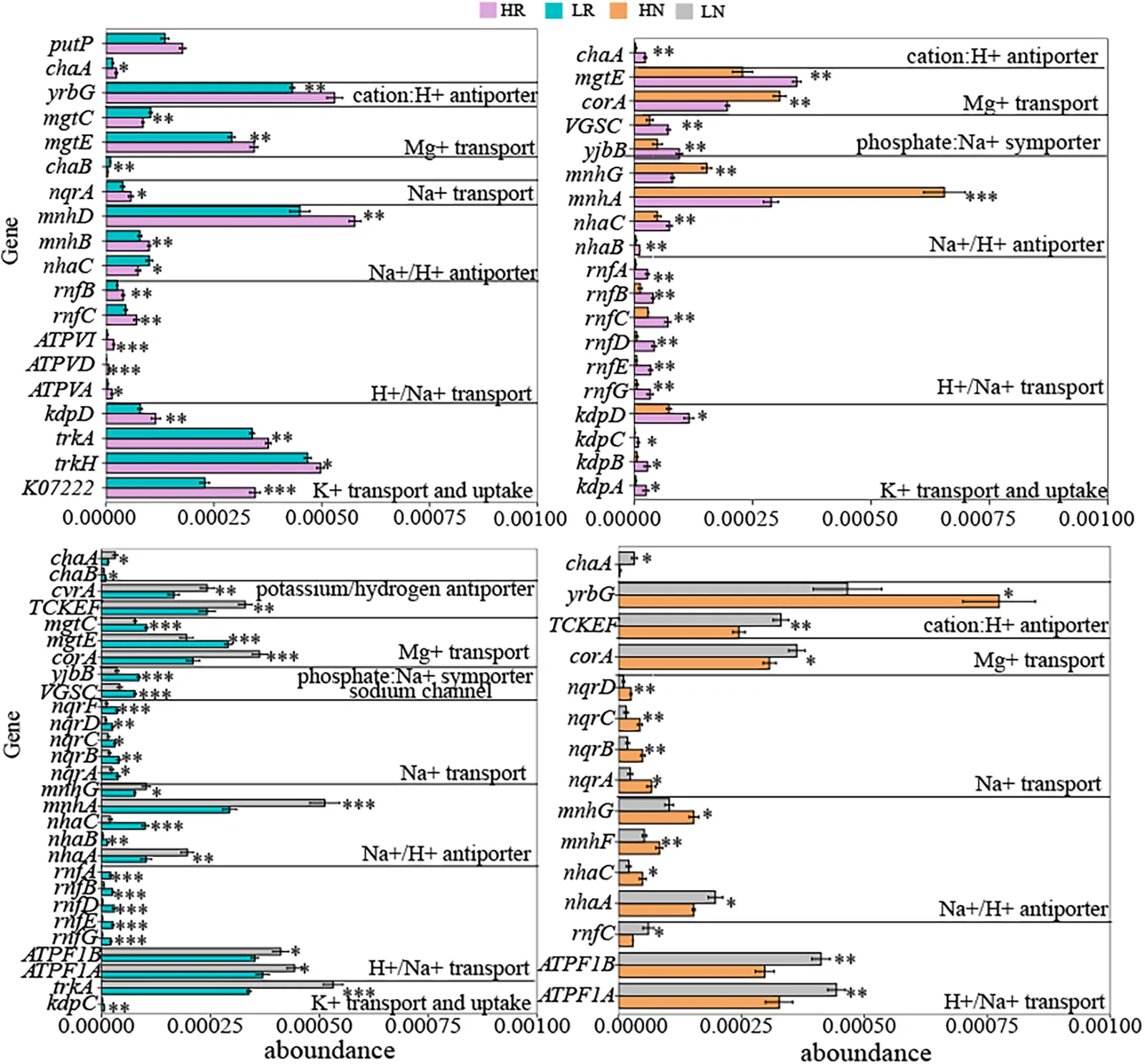
Differential genes of microbial ion transport in two saline habitats. * represents significant differences between different groups in α=0.05, ** represents significant differences between different groups in α=0.01, *** represents significant differences between different groups in α=0.001, the same as below.

The relative abundance of genes involved in glutamate synthesis by soil microorganisms (*GDH2*, *GLUD1_2*, *proA*, *proB*, *glmS*, *glnE*, *guaA*, *hemK*), genes involved in proline synthesis (*PRODH*, *pip*), genes involved in ectoine synthesis (*doeA*, *ectA*), and genes involved in trehalose synthesis (*otsB*) were all higher in the high salt rhizosphere than in the low salt rhizosphere. In bulk soil, there are fewer genes annotated for microbial involvement in osmotic regulation substances. The abundance of *TREH*, *betT*, *ectA*, and *ectC* genes were higher in high salt bulk soil than in low salt bulk soil, while the relative abundance changes of *glnA*, *gltB*, *otsA*, and *proP* were opposite (**Figure 6**).

**Figure 6:**
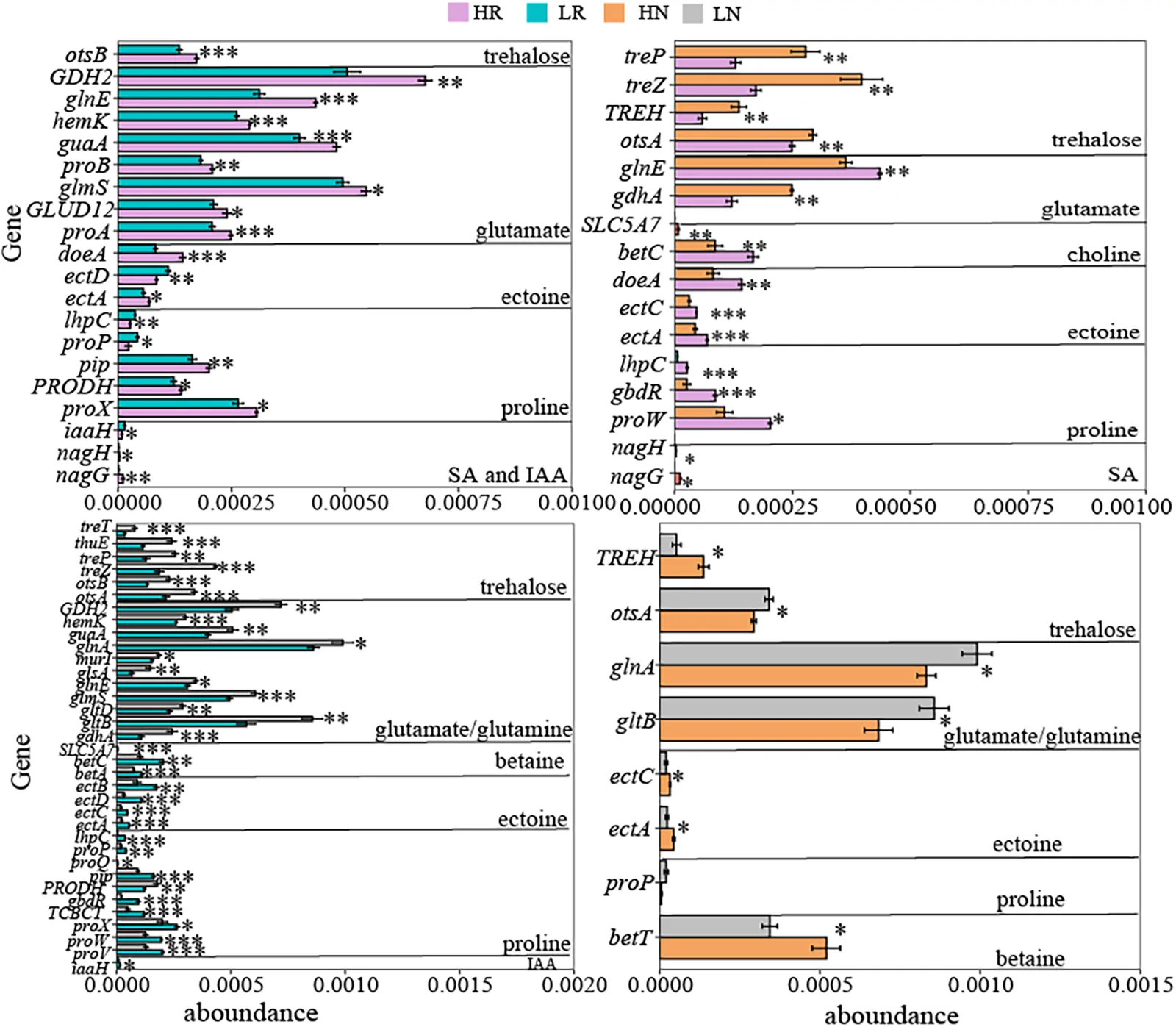
Differential genes of microbial osmoregulatory substances in two saline habitats. * *P* < 0.05, ** *P* < 0.01, *** *P* < 0.001.

### The network relationship between the abundance of dominant phyla and salt tolerant functional genes

Network analysis showed that there are more nodes and edges between dominant phylum microorganisms and ion transport functional genes in rhizosphere soil (34 and 91), compared to 26 and 62 in bulk soil. The correlation between dominant phyla microorganisms in rhizosphere and bulk soil and ion transport functional genes was mainly positive, accounting for about 62%. The dominant phylum microorganisms in rhizosphere soil were mainly positively correlated with functional genes related to osmotic regulation and hormones, while bulk microorganisms were mainly negatively correlated (**Figure 7**).

**Figure 7:**
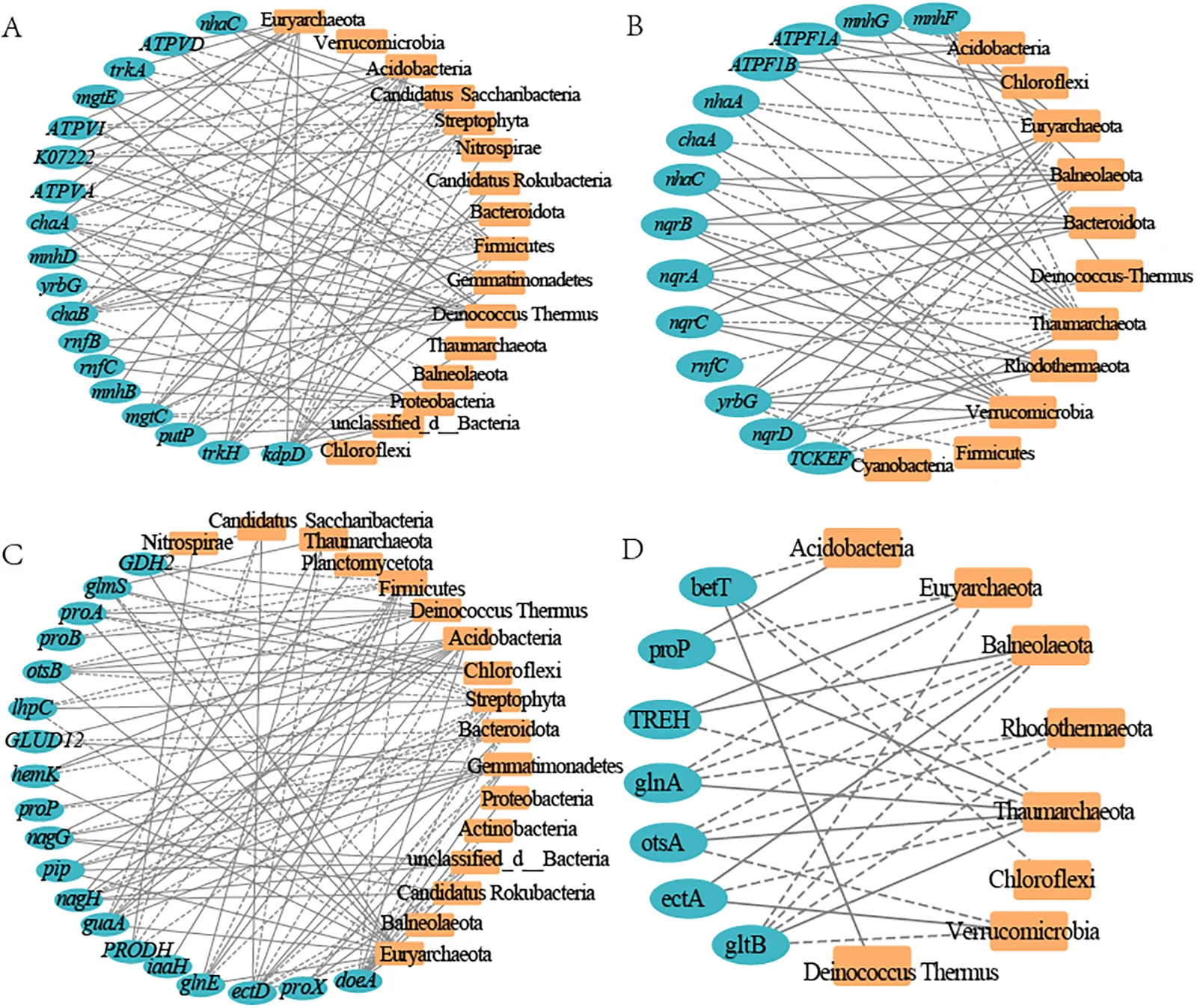
The correlation between the abundance of soil microbial phyla and the abundance of salt tolerant functional genes. **(A, B)** refers to the correlation between dominant phylum microorganisms and ion transport functional genes in rhizosphere and bulk soil, respectively; **(C, D)** refers to the correlation between dominant phyla microorganisms in rhizosphere and bulk soil and genes related to osmotic regulation.

### The influence of salt tolerant and halophilic microorganisms on the abundance of salt tolerant functional genes

Due to the high correlation between salt tolerant and halophilic microorganisms and the abundance of salt tolerant functional genes in the rhizosphere and bulk of *K. foliatum*, only results with a correlation coefficient greater than 0.8 were shown. There was a high correlation between halophilic archaea and the abundance of salt tolerant functional genes, and both were mainly positively correlated (**Figures 8**, **9**).

**Figure 8:**
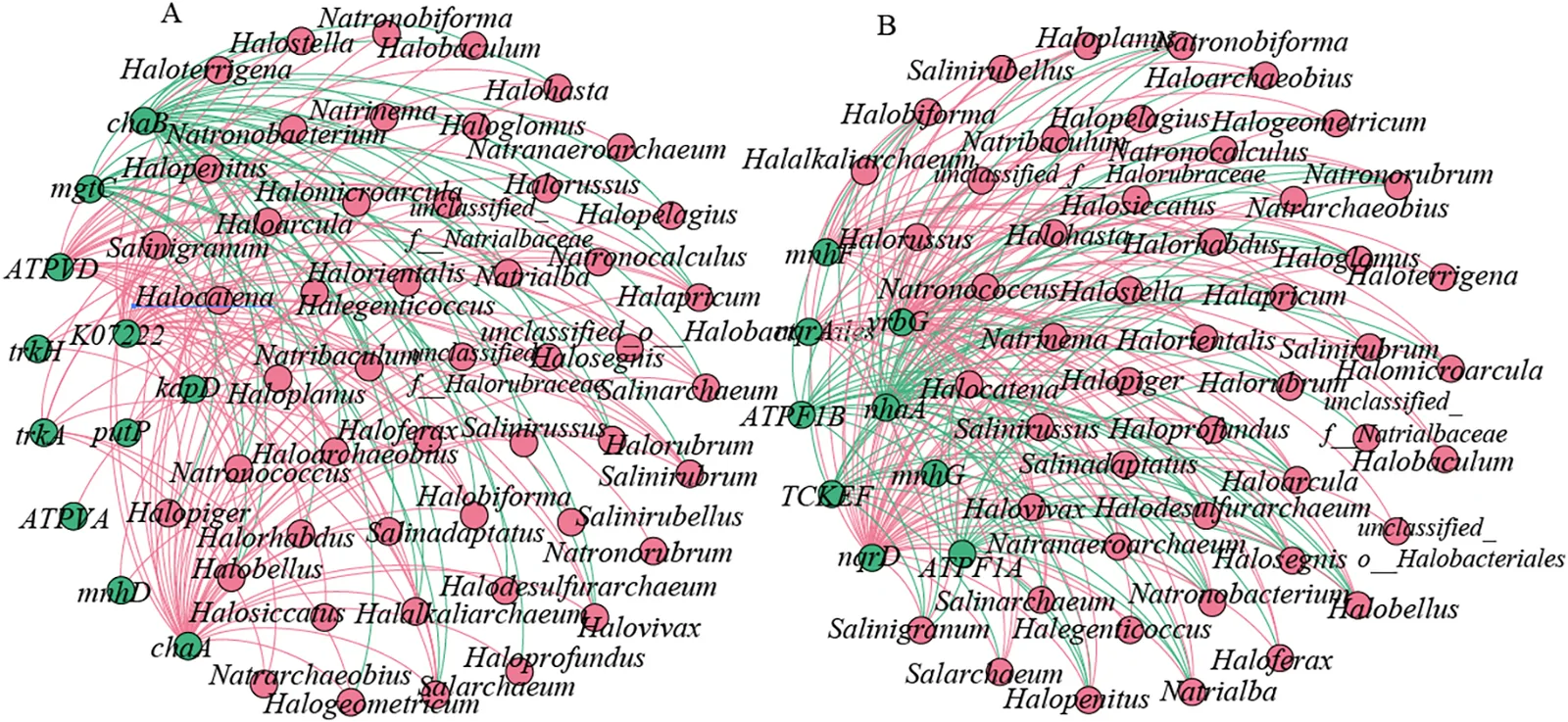
The correlation between the abundance of halophilic microorganisms and the abundance of salt tolerant functional genes related to ion transport functions. **(A, B)** represent rhizosphere soil and bulk soil. The red line represents positive correlation, and the green line represents negative correlation.

**Figure 9:**
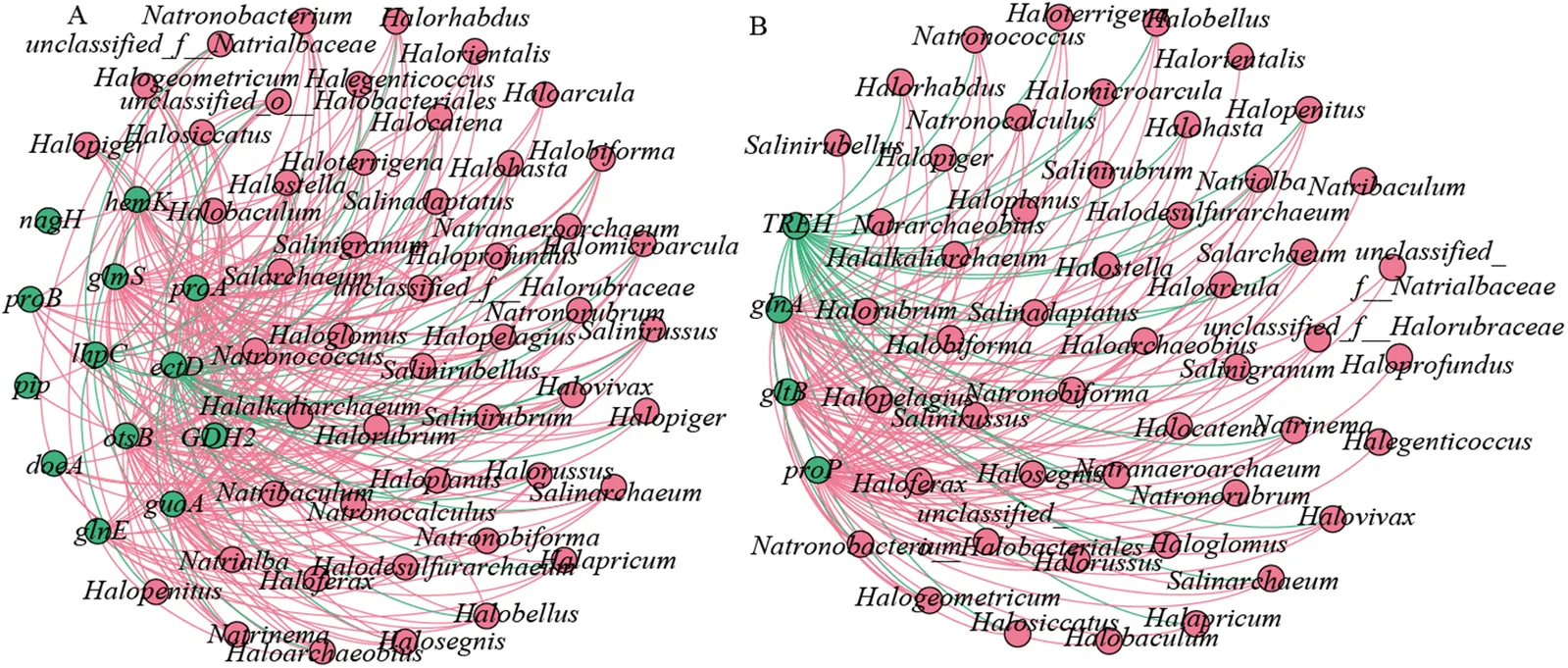
The correlation between the abundance of halophilic microorganisms and the abundance of salt tolerant functional genes related to osmotic regulation functions. **(A, B)** represent rhizosphere soil and bulk soil. The red line represents positive correlation, and the green line represents negative correlation.

## Discussion

The Chao index of soil microorganisms in the rhizosphere of *K. foliatum* showed that high salt habitat was greater than low salt habitat, while the Shannon index showed that low salt rhizosphere soil was significantly greater than low salt bulk soil (**Figure 2**). Under high salt conditions, the rhizosphere microbial community exhibits increased species richness but reduced evenness, as reflected by higher Chao and Simpson indices and a lower Shannon index, a pattern that suggests the selective enrichment of specialized halotolerant taxa capable of thriving under extreme osmotic stress. Actinobacteria play an important role in biogeochemical cycling and soil development. The report by Wang et al. (2016) indicates that actinomycetes are the most abundant halophilic bacteria in saline soil. Similarly, this study found similar results Overall, the decrease in Actinobacteria, increase in Bacteroidetes, and decrease in Chloroflexi in high salt soil habitat (**Figure 3**), indicate that high salt environments can disrupt the intracellular osmotic pressure balance of actinomycetes (Grishko et al., 2015), inhibit their hyphal growth and extracellular enzyme activity, resulting in a decrease in their numbers. The increase in Bacteroidetes in high salt habitat is functionally associated with an increase in the demand for stubborn carbon decomposition (Wu, 2025; Wang et al., 2025), The reduction in Chloroflexi abundance may reflect the loss of specific anaerobic or fermentative functions they perform, such as the degradation of complex organic compounds under anoxic conditions (Li et al., 2024).

Plants must face two main stresses under high salinity, namely ion stress and osmotic stress (Horie et al., 2012). However, in many cases, these mechanisms are insufficient to fully protect plant growth and development from significant inhibition. Fortunately, soil microorganisms have a positive impact on plant growth. The first method is to improve salt tolerance by regulating ion transport. High salt conditions are characterized by elevated extracellular Na^+^ concentrations that disrupt cellular ion homeostasis. In our study, the upregulation of *nqrA* and *nhaC* (Na^+^/H^+^ antiporter)、*mnhB/D* (**Figure 5**), indicated an enhanced capacity for Na^+^ efflux, driven by a proton motive force generated by ATP hydrolysis or electron transport. This mechanism is critical for maintaining a low cytosolic Na^+^ concentration, which prevents the disruption of enzymatic systems and protein synthesis. Simultaneously, the increased abundance of *rnfC* and *chaA* (Ca^2+^/H^+^ antiporter) suggested that the microbiome also regulated the homeostasis of other cations (Ca^2+^, Mg^2+^) that were essential for membrane stability and enzyme function. The upregulation of *trkA* and *trkH* (K^+^ uptake systems) reflects another key adaptation: maintaining a high intracellular K^+^ concentration is a well-established strategy to counteract Na^+^ toxicity, because K^+^ is the primary osmoticum in most bacterial cells. By increasing K^+^ uptake, the microbiome helps maintain an optimal K^+^/Na^+^ ratio, which is essential for preventing ionic toxicity and preserving metabolic activity (Almeida et al., 2017). The second approach is to play a key role in plant salt tolerance by generating osmoregulatory substances (Del Carmen et al., 2019), ectoine and betaine are amino acid derivatives that play a crucial role in cellular homeostasis. Under high-salt conditions, these compounds effectively reduce cytoplasmic water activity, thereby preserving the functionality of intracellular enzymes (Zeng et al., 2022). This study found significant differences in the synthesis genes of proline (*proX*), trehalose (*otsB*), and ectoine (*ectA*) small molecule osmoregulatory substances by *K. foliatum* rhizosphere microorganisms in high salt and low salt habitat, and the relative abundance of these genes was mostly higher in high salt rhizosphere than in low salt habitat (**Figure 6**). This indicates that the proline and other small molecule substances produced by microorganisms can serve as endogenous osmotic substances in plants, directly protect plants, or induce plant salt tolerance as signals (Del Carmen et al., 2019). In this study, it was found that the abundance of *ProDH* in high salt rhizosphere was lower than that in high salt bulk soil (**Figure 6**), indicating that high salt rhizosphere can alleviate plant damage caused by high salt stress by reducing *ProDH* abundance, decreasing its activity, clearing free radicals and protecting cell structure (Naveed et al., 2024).

There were abundant halophilic bacteria in *K. foliatum* rhizosphere in high salt habitat (**Figure 4**; **Supplementary Figures S1**, **S2**). Research has shown that there is an H^+^ pump in the cell membrane of halophilic bacteria, which endows these bacteria with the function of Na^+^ reverse transport, forcing salt ions to move from the inside of the cell to the outside (Plemenitaš et al., 2016). The halophilic bacteria cells can maintain a high internal concentration of K^+^ (Meng et al., 2019), which better explains the increase in Na^+^ antiporters and K^+^ absorption and transport functional genes in the rhizosphere soil microorganisms of *K. foliatum*. This adaptive mechanism is commonly exhibited by halophilic archaea and anaerobic halophilic bacteria. To mitigate elevated extracellular osmotic pressure, these microbes produce specific organic osmolytes. Interestingly, the types of accumulated osmolytes vary significantly with salt concentration (Lai and Lai, 2011; Lai et al., 2014). In this study, the key genes involved in betaine synthesis in the rhizosphere of *K. foliatum* did not significantly increase in high salt habitat, but the key genes involved in trehalose, ectoine, and proline synthesis significantly increased, indicating that different osmotic regulators have different salt tolerance (**Figure 6**). It may be related to the different metabolic mechanisms of halophilic microorganisms in response to external high salt stress. The abundance of genes (*nagH/G*) involved in the production of salicylic acid by *K. foliatum* rhizosphere microorganisms is significantly higher than that in low salt rhizosphere (**Figure 6**), indicating that plant growth promoting rhizobacteria can improve salt tolerance by producing salicylic acid. The network analysis results indicated that the dominant phylum microorganisms in the rhizosphere of *K. foliatum* mainly participate in ion transport and osmotic regulation through positive correlation, and the number of dominant phylum microorganisms involved in regulation in the rhizosphere was greater than that in the bulk soil (**Figure 7**), indicating a stronger collaborative ability of rhizosphere microorganisms. The correlation between halophilic microorganisms and ion transport and osmotic regulation functional genes indicated that halophilic archaea have a greater impact on functional genes in rhizosphere (**Figures 8**, **9**).

In recent years, multiple metagenomic studies have been published on the rhizosphere microbiome of halophilic plants along salinity gradients, providing valuable references for the results of this study. For example, a metagenomic analysis of the *Tamarix* rhizosphere microbiome along soil salinity gradients showed that higher salinity was accompanied by enrichment of genetic information processing and ion transport related functions, increasing the representativeness of the Na ^+^/H ^+^ antiporter system and V/A type ATPase (Wang et al., 2026). This is consistent with the upregulation of ion transport genes (*nqrA*, *nhaC*, *rnfC*, *chaA*, *yrbG*) observed in the high salt rhizosphere of K. *foliatum*. Similarly, a study using metagenomics to investigate the diversity of rhizosphere microbiota and salt stress relieving genes in coastal salt tolerant wild plants (*Suaeda glauca*, *Phragmites australis*, and *Spartina alterniflora*) found that salt stress-related pathways were more abundant in the core genera *Pseudomonas* and *Woeseia*. The results of this study mainly focused on functional genes in metabolic pathways such as glycolysis and ABC transporter phosphate solubilization (Li et al., 2026), and did not pay attention to changes in ion transport and osmotic regulation functional genes.

Notably, the ecological context of our study, the Ebinur Lake Wetland National Nature Reserve in Xinjiang, an extreme arid desert ecosystem, provides a distinctive setting that differs markedly from coastal wetlands, Mediterranean salt marshes, or experimentally salinized soils. This context offers a unique perspective on halophyte-microbiome adaptations under the combined pressures of aridity and salinity.

## Conclusion

In this study, we demonstrate that the rhizosphere microbiome of the halophyte *K. foliatum* is not merely a passive responder to soil salinity, but an active functional entity that orchestrates a coordinated salt-tolerance strategy through both community reassembly and gene regulation. Rhizosphere microbial community of *K. foliatum* undergoes a systematic restructuring along the salinity gradient, characterized by increased species richness but reduced evenness under high salt conditions (higher Chao and Simpson indices, lower Shannon index). This pattern indicates a process of ecological selection: high salinity imposes a strong environmental filter that eliminates salt-sensitive taxa while simultaneously enriching a subset of specialized halotolerant microorganisms. This compositional shift is accompanied by a systematic upregulation of genes governing ion transport (e.g., *nqrA*, *nhaC*, *rnfC*, *chaA*, and *yrbG*) and compatible solute synthesis (e.g., *prop* and *ectA*), indicating that the microbiome jointly reinforces cellular ion homeostasis and osmotic balance to mitigate salt injury. Critically, correlation network analyses pinpoint halophilic archaea as the dominant drivers of these functional gene expressions, surpassing other microbial groups in their regulatory influence, a finding that underscores their keystone role in the plant-microbe salt-adaptive interaction. However, as this study was based metagenome sequencing data, the actual activities of these genes and their regulatory mechanisms require experimental validation through culture-based assays or targeted genetic manipulation in future work.

## Data Availability

The sequencing data have been deposited in the National Center for Biotechnology Information Sequence Read Archive database (http://www.ncbi.nlm.nih.gov/sra) with BioProject accession number PRJNA1405899.
