# Supplementary material for: Differences in microbial composition and salt tolerance between rhizosphere and bulk soil of Kalidium foliatum
Source: Front Plant Sci. 2026 Oct 7;17:1920612. doi: 10.3389/fpls.2026.1920612 (PMC13683641; doi:10.3389/fpls.2026.1920612)
Supplement: Supplementary file 1 [file DataSheet1.docx]

Differences in microbial composition and salt tolerance between rhizosphere and bulk soil of *Kalidium foliatum*

Figure S1 Differences in the abundance of halophilic archaea in the rhizosphere soil of *K. foliatum* under different habitats (Continued Figure 4)

Figure S2 Differences in the abundance of halophilic archaea in the rhizosphere soil of *K. foliatum* under different habitats (Continued Figure 4)
